# Dimethyl 5,6,7-trimeth­oxy-2-methyl-1,2-dihydro­quinoline-2,4-dicarboxyl­ate

**DOI:** 10.1107/S1600536811004028

**Published:** 2011-02-05

**Authors:** Zeynep Gültekin, Wolfgang Frey, Tuncer Hökelek

**Affiliations:** a Department of Chemistry, Çankırı Karatekin University, TR-18100 Çankırı, Turkey; b Universitat Stuttgart, Pfaffenwaldring 55, D-70569 Stuttgart, Germany; c Department of Physics, Hacettepe University, 06800 Beytepe Ankara, Turkey

## Abstract

In the title compound, C_17_H_21_NO_7_, the dihydro­pyridine ring assumes a screw-boat conformation. In the crystal, inter­molecular C—H⋯O hydrogen bonds link the mol­ecules, forming supra­molecular chains running along the *b* axis.

## Related literature

For the preparation of 1,2-dihydro­quinoline, see: Edwards *et al.* (1998 ▶); Yan *et al.* (2004 ▶); Petasis & Butkevich (2009 ▶); Johnson *et al.* (1989 ▶); Gültekin *et al.* (2010 ▶); Waldmann *et al.* (2008 ▶). For the biological activity of dihydro­quinolines, see: Elmore *et al.* (2001 ▶); Dillard *et al.* (1973 ▶); Muren & Weissmann (1971 ▶). For ring puckering parameters, see: Cremer & Pople (1975 ▶).
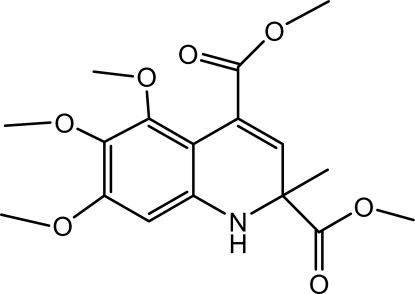


## Experimental

### 

#### Crystal data

C_17_H_21_NO_7_
*M* *_r_* = 351.35
Orthorhombic, 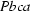

*a* = 10.476 (2) Å
*b* = 16.552 (4) Å
*c* = 20.238 (4) Å
*V* = 3509.2 (13) Å^3^
*Z* = 8
Mo *K*α radiation
μ = 0.10 mm^−1^
*T* = 294 K
0.6 × 0.4 × 0.15 mm

#### Data collection

Nicolet P3 diffractometer
3447 measured reflections
3447 independent reflections
1839 reflections with *I* > 2σ(*I*)
3 standard reflections every 50 reflections  intensity decay: 1%

#### Refinement

*R*[*F* ^2^ > 2σ(*F* ^2^)] = 0.070
*wR*(*F* ^2^) = 0.156
*S* = 1.07
3447 reflections
237 parameters
H atoms treated by a mixture of independent and constrained refinement
Δρ_max_ = 0.18 e Å^−3^
Δρ_min_ = −0.17 e Å^−3^

### 

Data collection: *XSCANS* (Siemens, 1996 ▶); cell refinement: *XSCANS*; data reduction: *SHELXTL* (Sheldrick, 2008 ▶); program(s) used to solve structure: *SHELXS97* (Sheldrick, 2008 ▶); program(s) used to refine structure: *SHELXL97* (Sheldrick, 2008 ▶); molecular graphics: *ORTEP-3 for Windows* (Farrugia, 1997 ▶); software used to prepare material for publication: *WinGX* (Farrugia, 1999 ▶).

## Supplementary Material

Crystal structure: contains datablocks I, global. DOI: 10.1107/S1600536811004028/xu5153sup1.cif

Structure factors: contains datablocks I. DOI: 10.1107/S1600536811004028/xu5153Isup2.hkl

Additional supplementary materials:  crystallographic information; 3D view; checkCIF report

## Figures and Tables

**Table 1:** Hydrogen-bond geometry (Å, °)

*D*—H⋯*A*	*D*—H	H⋯*A*	*D*⋯*A*	*D*—H⋯*A*
C14—H14*A*⋯O1^i^	0.96	2.51	3.251 (6)	134

Symmetry code: (i) 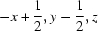.

**Fig. 1.**
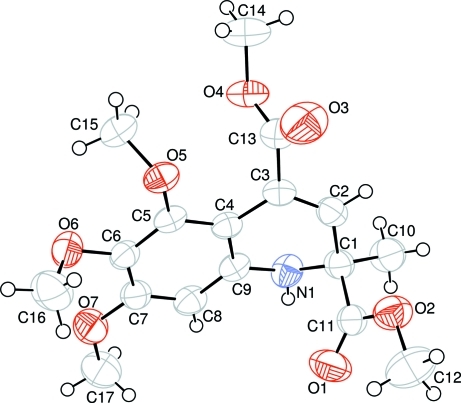
The molecular structure of the title compound with the atom-numbering scheme. Displacement ellipsoids are drawn at the 50% probability level.

**Fig. 2.**
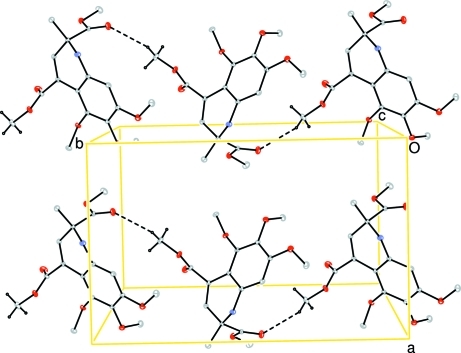
A partial packing diagram viewed down the c-axis. Hydrogen bonds are shown as dashed lines.

## supplementary crystallographic information

### Comment

Dihydroquinolines have been widely studied and found an important structural unit in synthetic organic and medicinal chemistry (Elmore *et al.*, 2001; Dillard *et al.*, 1973; Muren & Weissmann, 1971). Many dihydroquinoline derivatives have been reported in the literature (Edwards *et al.*, 1998; Yan *et al.*, 2004; Petasis & Butkevich, 2009; Gültekin *et al.*, 2010) and some of them have biological effects. For example, 2,2,4-substituted 1,2-dihydroquinolines have been shown antibacterial activities (Johnson *et al.*, 1989).

In the title compound, (I), (Fig. 1), the ring A (C1-C4/C9/N1) is not planar; the puckering parameters (Cremer & Pople, 1975) Q_T_ = 0.379 (3) Å, φ = 21.5 (6)° and θ = 66.4 (5)° suggesting a screw-boat conformation. In the crystal structure, intermolecular C-H···O hydrogen bonds (Table 1) link the molecules to form infinite chains along the b-axis (Fig. 2).

### Experimental

The title compound was synthesized by the literature method (Waldmann *et al.*, 2008). 3,4,5-dimethoxyaniline (100 mg, 1 eq) was dissolved in chloroform (1.5 ml) in a screw-capped test tube and Bi(OTf)_3_ (5 mol%, 0.05 eq) was added to the mixture. The mixture was stirred at room temperature for 4 h until the starting material was completely consumed as monitored by TLC. The resultant residue was directly purified by flash chromatography on silica (EtOAc:Cylohexane 2:98) gave in 83% yield as a yellow solid. Recrystallized over pentane and ethyl acetate (70:30) gave yellow crystalline solid R_f_ 0.16 (2:1 Cyclohexane/EtOAc) mp 394-395 K.

### Crystal data

**Table:**

C_17_H_21_NO_7_	*F*(000) = 1488
*M**_r_* = 351.35	*D*_x_ = 1.330 Mg m^−^^3^
Orthorhombic, *P**b**c**a*	Mo *K*α radiation, λ = 0.71073 Å
Hall symbol: -P 2ac 2ab	Cell parameters from 40 reflections
*a* = 10.476 (2) Å	θ = 10–12°
*b* = 16.552 (4) Å	µ = 0.10 mm^−^^1^
*c* = 20.238 (4) Å	*T* = 294 K
*V* = 3509.2 (13) Å^3^	Plates, colourless
*Z* = 8	0.6 × 0.4 × 0.15 mm

### Data collection

**Table:**

Nicolet P3 diffractometer	*R*_int_ = 0.000
Radiation source: fine-focus sealed tube	θ_max_ = 26.0°, θ_min_ = 2.0°
graphite	*h* = 0→12
Wyckoff scan	*k* = 0→20
3447 measured reflections	*l* = 0→24
3447 independent reflections	3 standard reflections every 50 reflections
1839 reflections with *I* > 2σ(*I*)	intensity decay: 1%

### Refinement

**Table:**

Refinement on *F*^2^	Secondary atom site location: difference Fourier map
Least-squares matrix: full	Hydrogen site location: inferred from neighbouring sites
*R*[*F*^2^ > 2σ(*F*^2^)] = 0.070	H atoms treated by a mixture of independent and constrained refinement
*wR*(*F*^2^) = 0.156	*w* = 1/[σ^2^(*F*_o_^2^) + (0.0359*P*)^2^ + 3.112*P*] where *P* = (*F*_o_^2^ + 2*F*_c_^2^)/3
*S* = 1.07	(Δ/σ)_max_ < 0.001
3447 reflections	Δρ_max_ = 0.18 e Å^−^^3^
237 parameters	Δρ_min_ = −0.17 e Å^−^^3^
0 restraints	Extinction correction: *SHELXL97* (Sheldrick, 2008), Fc^*^=kFc[1+0.001xFc^2^λ^3^/sin(2θ)]^-1/4^
Primary atom site location: structure-invariant direct methods	Extinction coefficient: 0.0031 (3)

### Special details

**Table:**

Geometry. All esds (except the esd in the dihedral angle between two l.s. planes) are estimated using the full covariance matrix. The cell esds are taken into account individually in the estimation of esds in distances, angles and torsion angles; correlations between esds in cell parameters are only used when they are defined by crystal symmetry. An approximate (isotropic) treatment of cell esds is used for estimating esds involving l.s. planes.
Refinement. Refinement of F^2^ against ALL reflections. The weighted R-factor wR and goodness of fit S are based on F^2^, conventional R-factors R are based on F, with F set to zero for negative F^2^. The threshold expression of F^2^ > 2sigma(F^2^) is used only for calculating R-factors(gt) etc. and is not relevant to the choice of reflections for refinement. R-factors based on F^2^ are statistically about twice as large as those based on F, and R- factors based on ALL data will be even larger.

### Fractional atomic coordinates and isotropic or equivalent isotropic displacement parameters (Å^2^)

**Table:**

	*x*	*y*	*z*	*U*_iso_*/*U*_eq_	
O1	0.5908 (3)	1.03546 (16)	0.62717 (15)	0.0881 (10)	
O2	0.6233 (3)	0.93649 (16)	0.55547 (13)	0.0713 (8)	
O3	0.2547 (3)	0.7747 (2)	0.51994 (16)	0.1002 (11)	
O4	0.1503 (3)	0.75844 (14)	0.61564 (14)	0.0745 (8)	
O5	0.0584 (2)	0.91803 (14)	0.58046 (11)	0.0540 (6)	
O6	−0.0366 (2)	1.05816 (15)	0.63970 (12)	0.0637 (7)	
O7	0.1046 (3)	1.13852 (16)	0.72647 (13)	0.0736 (8)	
N1	0.4420 (3)	0.9400 (2)	0.70665 (15)	0.0590 (9)	
H21	0.485 (4)	0.973 (2)	0.733 (2)	0.091 (16)*	
C1	0.5191 (3)	0.9018 (2)	0.65584 (19)	0.0525 (9)	
C2	0.4333 (3)	0.8467 (2)	0.61660 (18)	0.0536 (9)	
H2	0.4696	0.8031	0.5946	0.064*	
C3	0.3086 (3)	0.85768 (19)	0.61218 (16)	0.0479 (8)	
C4	0.2492 (3)	0.92839 (19)	0.64312 (15)	0.0445 (8)	
C5	0.1281 (3)	0.9578 (2)	0.62794 (16)	0.0457 (8)	
C6	0.0824 (3)	1.0285 (2)	0.65599 (17)	0.0489 (9)	
C7	0.1567 (3)	1.0694 (2)	0.70186 (17)	0.0541 (9)	
C8	0.2776 (3)	1.0412 (2)	0.71861 (17)	0.0566 (10)	
H8	0.3272	1.0688	0.7493	0.068*	
C9	0.3233 (3)	0.9712 (2)	0.68871 (16)	0.0489 (9)	
C10	0.6265 (4)	0.8538 (2)	0.6884 (2)	0.0730 (12)	
H10A	0.6801	0.8899	0.7132	0.109*	
H10B	0.6763	0.8274	0.6550	0.109*	
H10C	0.5907	0.8141	0.7176	0.109*	
C11	0.5798 (3)	0.9663 (2)	0.61161 (19)	0.0528 (9)	
C12	0.6849 (5)	0.9931 (3)	0.5108 (2)	0.1013 (16)	
H12A	0.6974	0.9678	0.4686	0.152*	
H12B	0.7660	1.0088	0.5287	0.152*	
H12C	0.6319	1.0400	0.5055	0.152*	
C13	0.2345 (4)	0.7940 (2)	0.5758 (2)	0.0613 (10)	
C14	0.0782 (5)	0.6925 (3)	0.5864 (3)	0.118 (2)	
H14A	0.0260	0.6676	0.6197	0.177*	
H14B	0.1362	0.6532	0.5686	0.177*	
H14C	0.0249	0.7130	0.5517	0.177*	
C15	−0.0622 (3)	0.8868 (2)	0.6031 (2)	0.0755 (12)	
H15A	−0.1022	0.8569	0.5682	0.113*	
H15B	−0.1164	0.9308	0.6160	0.113*	
H15C	−0.0483	0.8519	0.6403	0.113*	
C16	−0.0349 (5)	1.1165 (3)	0.5882 (2)	0.0906 (15)	
H16A	−0.1179	1.1406	0.5842	0.136*	
H16B	−0.0124	1.0907	0.5474	0.136*	
H16C	0.0268	1.1576	0.5984	0.136*	
C17	0.1819 (4)	1.1883 (2)	0.7671 (2)	0.0896 (15)	
H17A	0.1360	1.2367	0.7781	0.134*	
H17B	0.2587	1.2022	0.7438	0.134*	
H17C	0.2033	1.1597	0.8069	0.134*	

### Atomic displacement parameters (Å^2^)

**Table:**

	*U*^11^	*U*^22^	*U*^33^	*U*^12^	*U*^13^	*U*^23^
O1	0.111 (3)	0.0508 (17)	0.103 (2)	−0.0080 (17)	0.014 (2)	0.0001 (16)
O2	0.0779 (19)	0.0685 (17)	0.0676 (18)	−0.0126 (15)	0.0092 (15)	−0.0002 (15)
O3	0.101 (2)	0.115 (3)	0.085 (2)	−0.028 (2)	0.012 (2)	−0.047 (2)
O4	0.0813 (18)	0.0507 (15)	0.092 (2)	−0.0248 (15)	−0.0035 (17)	−0.0025 (15)
O5	0.0507 (14)	0.0598 (15)	0.0516 (14)	−0.0123 (12)	−0.0065 (12)	−0.0034 (12)
O6	0.0493 (15)	0.0704 (17)	0.0712 (18)	0.0042 (13)	0.0024 (13)	−0.0002 (14)
O7	0.0750 (19)	0.0670 (17)	0.0789 (19)	0.0009 (15)	0.0051 (15)	−0.0253 (15)
N1	0.0520 (19)	0.073 (2)	0.0524 (19)	−0.0034 (18)	−0.0093 (16)	−0.0077 (17)
C1	0.050 (2)	0.048 (2)	0.060 (2)	0.0010 (18)	−0.0078 (18)	0.0047 (18)
C2	0.056 (2)	0.0443 (19)	0.060 (2)	0.0016 (18)	−0.0018 (19)	0.0009 (17)
C3	0.055 (2)	0.0426 (19)	0.046 (2)	−0.0088 (17)	−0.0019 (17)	0.0038 (16)
C4	0.0478 (18)	0.0465 (18)	0.0391 (17)	−0.0091 (17)	0.0019 (16)	−0.0005 (16)
C5	0.0453 (19)	0.052 (2)	0.0395 (18)	−0.0142 (17)	−0.0014 (16)	−0.0009 (16)
C6	0.0425 (19)	0.053 (2)	0.051 (2)	−0.0064 (17)	0.0045 (17)	−0.0020 (17)
C7	0.055 (2)	0.052 (2)	0.055 (2)	−0.0057 (19)	0.0102 (19)	−0.0077 (18)
C8	0.058 (2)	0.063 (2)	0.049 (2)	−0.013 (2)	0.0029 (18)	−0.0144 (19)
C9	0.047 (2)	0.056 (2)	0.043 (2)	−0.0094 (18)	−0.0022 (17)	0.0025 (17)
C10	0.064 (2)	0.069 (3)	0.086 (3)	0.003 (2)	−0.017 (2)	0.020 (2)
C11	0.045 (2)	0.050 (2)	0.063 (2)	0.0026 (18)	−0.0061 (19)	0.0045 (19)
C12	0.104 (4)	0.115 (4)	0.085 (3)	−0.029 (3)	0.017 (3)	0.027 (3)
C13	0.060 (2)	0.054 (2)	0.070 (3)	−0.008 (2)	−0.001 (2)	−0.009 (2)
C14	0.106 (4)	0.072 (3)	0.176 (6)	−0.041 (3)	−0.005 (4)	−0.029 (3)
C15	0.050 (2)	0.075 (3)	0.102 (3)	−0.016 (2)	−0.006 (2)	−0.009 (2)
C16	0.094 (3)	0.072 (3)	0.106 (4)	0.006 (3)	−0.017 (3)	0.021 (3)
C17	0.104 (4)	0.073 (3)	0.092 (4)	−0.006 (3)	0.000 (3)	−0.038 (3)

### Geometric parameters (Å, °)

**Table:**

O1—C11	1.193 (4)	C5—C6	1.386 (5)
O2—C11	1.320 (4)	C6—C7	1.387 (5)
O2—C12	1.453 (5)	C7—C8	1.391 (5)
O3—C13	1.194 (4)	C8—C9	1.393 (5)
O4—C13	1.332 (4)	C8—H8	0.9300
O4—C14	1.453 (5)	C10—H10A	0.9600
O5—C5	1.374 (4)	C10—H10B	0.9600
O5—C15	1.440 (4)	C10—H10C	0.9600
O6—C6	1.380 (4)	C12—H12A	0.9600
O6—C16	1.421 (5)	C12—H12B	0.9600
O7—C7	1.362 (4)	C12—H12C	0.9600
O7—C17	1.418 (4)	C14—H14A	0.9600
N1—C1	1.453 (4)	C14—H14B	0.9600
N1—C9	1.394 (4)	C14—H14C	0.9600
N1—H21	0.89 (4)	C15—H15A	0.9600
C1—C2	1.507 (5)	C15—H15B	0.9600
C1—C10	1.527 (5)	C15—H15C	0.9600
C1—C11	1.532 (5)	C16—H16A	0.9600
C2—C3	1.322 (4)	C16—H16B	0.9600
C2—H2	0.9300	C16—H16C	0.9600
C3—C4	1.466 (5)	C17—H17A	0.9600
C3—C13	1.502 (5)	C17—H17B	0.9600
C4—C5	1.393 (5)	C17—H17C	0.9600
C4—C9	1.399 (4)		
			
C11—O2—C12	116.6 (3)	C1—C10—H10C	109.5
C13—O4—C14	115.5 (4)	H10A—C10—H10B	109.5
C5—O5—C15	114.5 (3)	H10A—C10—H10C	109.5
C6—O6—C16	113.9 (3)	H10B—C10—H10C	109.5
C7—O7—C17	118.1 (3)	O1—C11—O2	123.6 (4)
C1—N1—H21	114 (3)	O1—C11—C1	123.7 (4)
C9—N1—C1	118.2 (3)	O2—C11—C1	112.7 (3)
C9—N1—H21	112 (3)	O2—C12—H12A	109.5
N1—C1—C2	107.7 (3)	O2—C12—H12B	109.5
N1—C1—C10	109.3 (3)	O2—C12—H12C	109.5
N1—C1—C11	109.9 (3)	H12A—C12—H12B	109.5
C2—C1—C10	110.7 (3)	H12A—C12—H12C	109.5
C2—C1—C11	111.2 (3)	H12B—C12—H12C	109.5
C10—C1—C11	108.0 (3)	O3—C13—O4	124.9 (4)
C1—C2—H2	118.6	O3—C13—C3	124.1 (4)
C3—C2—C1	122.8 (3)	O4—C13—C3	110.9 (3)
C3—C2—H2	118.6	O4—C14—H14A	109.5
C2—C3—C4	120.1 (3)	O4—C14—H14B	109.5
C2—C3—C13	116.6 (3)	O4—C14—H14C	109.5
C4—C3—C13	123.4 (3)	H14A—C14—H14B	109.5
C5—C4—C3	124.9 (3)	H14A—C14—H14C	109.5
C5—C4—C9	118.3 (3)	H14B—C14—H14C	109.5
C9—C4—C3	116.7 (3)	O5—C15—H15A	109.5
O5—C5—C4	118.1 (3)	O5—C15—H15B	109.5
O5—C5—C6	120.5 (3)	O5—C15—H15C	109.5
C6—C5—C4	121.3 (3)	H15A—C15—H15B	109.5
O6—C6—C5	120.9 (3)	H15A—C15—H15C	109.5
O6—C6—C7	119.6 (3)	H15B—C15—H15C	109.5
C5—C6—C7	119.5 (3)	O6—C16—H16A	109.5
O7—C7—C6	115.4 (3)	O6—C16—H16B	109.5
O7—C7—C8	123.9 (3)	O6—C16—H16C	109.5
C6—C7—C8	120.7 (3)	H16A—C16—H16B	109.5
C7—C8—C9	119.1 (3)	H16A—C16—H16C	109.5
C7—C8—H8	120.5	H16B—C16—H16C	109.5
C9—C8—H8	120.5	O7—C17—H17A	109.5
N1—C9—C4	118.7 (3)	O7—C17—H17B	109.5
C8—C9—N1	120.1 (3)	O7—C17—H17C	109.5
C8—C9—C4	121.1 (3)	H17A—C17—H17B	109.5
C1—C10—H10A	109.5	H17A—C17—H17C	109.5
C1—C10—H10B	109.5	H17B—C17—H17C	109.5
			
C12—O2—C11—O1	−1.3 (6)	C2—C3—C4—C9	−14.3 (5)
C12—O2—C11—C1	−178.8 (3)	C13—C3—C4—C5	−19.0 (5)
C14—O4—C13—O3	−1.0 (6)	C13—C3—C4—C9	164.4 (3)
C14—O4—C13—C3	−176.6 (3)	C2—C3—C13—O3	−57.5 (6)
C15—O5—C5—C4	120.6 (3)	C2—C3—C13—O4	118.1 (4)
C15—O5—C5—C6	−64.2 (4)	C4—C3—C13—O3	123.8 (5)
C16—O6—C6—C5	−94.5 (4)	C4—C3—C13—O4	−60.6 (4)
C16—O6—C6—C7	86.5 (4)	C3—C4—C5—O5	−0.1 (5)
C17—O7—C7—C6	−172.3 (3)	C3—C4—C5—C6	−175.2 (3)
C17—O7—C7—C8	5.6 (5)	C9—C4—C5—O5	176.5 (3)
C9—N1—C1—C2	−45.4 (4)	C9—C4—C5—C6	1.3 (5)
C9—N1—C1—C10	−165.7 (3)	C3—C4—C9—N1	−6.3 (4)
C9—N1—C1—C11	76.0 (4)	C3—C4—C9—C8	177.2 (3)
C1—N1—C9—C8	−145.0 (3)	C5—C4—C9—N1	176.8 (3)
C1—N1—C9—C4	38.5 (5)	C5—C4—C9—C8	0.3 (5)
N1—C1—C2—C3	24.7 (5)	O5—C5—C6—O6	3.6 (5)
C10—C1—C2—C3	144.2 (4)	O5—C5—C6—C7	−177.4 (3)
C11—C1—C2—C3	−95.8 (4)	C4—C5—C6—O6	178.7 (3)
N1—C1—C11—O1	18.8 (5)	C4—C5—C6—C7	−2.3 (5)
N1—C1—C11—O2	−163.7 (3)	C5—C6—C7—O7	179.6 (3)
C2—C1—C11—O1	138.0 (4)	C5—C6—C7—C8	1.7 (5)
C2—C1—C11—O2	−44.5 (4)	O6—C6—C7—O7	−1.4 (5)
C10—C1—C11—O1	−100.4 (4)	O6—C6—C7—C8	−179.3 (3)
C10—C1—C11—O2	77.1 (4)	O7—C7—C8—C9	−177.8 (3)
C1—C2—C3—C4	3.5 (5)	C6—C7—C8—C9	0.0 (5)
C1—C2—C3—C13	−175.2 (3)	C7—C8—C9—N1	−177.4 (3)
C2—C3—C4—C5	162.3 (3)	C7—C8—C9—C4	−1.0 (5)

### Hydrogen-bond geometry (Å, °)

**Table:**

*D*—H···*A*	*D*—H	H···*A*	*D*···*A*	*D*—H···*A*
C14—H14A···O1^i^	0.96	2.51	3.251 (6)	134

Symmetry codes: (i) −*x*+1/2, *y*−1/2, *z*.
